# Examining Rural and Racial Disparities in the Relationship Between Loneliness and Social Technology Use Among Older Adults

**DOI:** 10.3389/fpubh.2021.723925

**Published:** 2021-08-31

**Authors:** Kaileigh A. Byrne, Reza Ghaiumy Anaraky, Cheryl Dye, Lesley A. Ross, Kapil Chalil Madathil, Bart Knijnenburg, Sue Levkoff

**Affiliations:** ^1^Department of Psychology, Clemson University, Clemson, SC, United States; ^2^Department of Human-Centered Computing, Clemson University, Clemson, SC, United States; ^3^Department of Civil Engineering, Clemson University, Clemson, SC, United States; ^4^Department of Industrial Engineering, Clemson University, Clemson, SC, United States; ^5^College of Social Work, University of South Carolina, Columbia, SC, United States

**Keywords:** aging, loneliness, technology, rurality, disparities

## Abstract

Loneliness, the subjective negative experience derived from a lack of meaningful companionship, is associated with heightened vulnerability to adverse health outcomes among older adults. Social technology affords an opportunity to cultivate social connectedness and mitigate loneliness. However, research examining potential inequalities in loneliness is limited. This study investigates racial and rural-urban differences in the relationship between social technology use and loneliness in adults aged 50 and older using data from the 2016 wave of the Health and Retirement Study (*N* = 4,315). Social technology use was operationalized as the self-reported frequency of communication through Skype, Facebook, or other social media with family and friends. Loneliness was assessed using the UCLA Loneliness scale, and rural-urban differences were based on Beale rural-urban continuum codes. Examinations of race focused on differences between Black/African-American and White/Caucasian groups. A path model analysis was performed to assess whether race and rurality moderated the relationship between social technology use and loneliness, adjusting for living arrangements, age, general computer usage. Social engagement and frequency of social contact with family and friends were included as mediators. The primary study results demonstrated that the association between social technology use and loneliness differed by rurality, but not race. Rural older adults who use social technology less frequently experience greater loneliness than urban older adults. This relationship between social technology and loneliness was mediated by social engagement and frequency of social contact. Furthermore, racial and rural-urban differences in social technology use demonstrated that social technology use is less prevalent among rural older adults than urban and suburban-dwelling older adults; no such racial differences were observed. However, Black older adults report greater levels of perceived social negativity in their relationships compared to White older adults. Interventions seeking to address loneliness using social technology should consider rural and racial disparities.

## Introduction

Loneliness is a significant public health problem associated with poor physical and mental health outcomes (1). The prevalence of loneliness has more than doubled over the last 40 years. In the late 1970s, only 11–17% of middle-aged and older adults reported experiencing loneliness (2, 3), yet a recent report showed that over a third of Americans aged 45 and older report experiencing loneliness (4). With advancing age, the prevalence of loneliness also increases−43% of older adults aged 65 and older report feeling lonely (5). Given the prevalence and detrimental consequences of loneliness, it is crucial to examine interventions and tools that may mitigate loneliness, particularly among older adults. Recent findings suggest that low levels of loneliness are associated with high levels of internet-based social technology use among individuals aged 65 and older (6). Therefore, social technology may be a helpful tool that can be leveraged to address the pervasiveness of loneliness among older adults. However, there are numerous potential barriers to equitable access to technology, and disparities in the interaction between loneliness and technology remain unclear. Consequently, the purpose of this study is to bridge this gap by examining rural-urban differences and racial differences between Blacks/African-Americans and Whites/Caucasians in the relationship between social technology use and loneliness.

The construct of loneliness can be defined as the perceived lack of close and meaningful social relationships (7, 8). A related yet distinctive concept is social isolation, which refers to having few social contacts and social connections (7–9). Thus, social isolation is the objective absence of others in one's social milieu, and loneliness is the subjective negative feeling of being psychologically distant from others. While social isolation increases the likelihood that an individual will feel lonely, it is not necessarily a prerequisite for the experience of loneliness (2). For example, individuals with a large social network can still experience loneliness if they do not find sufficient close, meaningful connections in their network. On the other hand, socially isolated individuals who have a few meaningful, supportive relationships may find those connections sufficient to not feel lonely (2, 8, 10). Thus, the quality of one's relationships, rather than quantity, influences feelings of loneliness (11).

One of the key rationales for understanding loneliness and identifying ways to alleviate it is that loneliness is linked to a heightened risk of numerous health problems. Loneliness among middle-aged adults is associated with a 26% increased likelihood of mortality—a rate that is comparable to the individual mortality risks of cigarette smoking, obesity, and substance abuse (12). Loneliness can decrease one's quality of life. Longitudinal studies have demonstrated that lonely older adults are more likely to experience rapid declines in physical functioning, including activities of daily living and mobility (5, 13, 14). The detrimental health correlates of loneliness further extend to cognitive functioning and mental health. Loneliness has been linked with a heightened risk of cognitive decline and dementia (15–18). In terms of mental health, several studies have shown evidence that loneliness is associated with higher rates of depression and anxiety among middle-aged and older adults (10, 19–24). Collectively, numerous studies have provided unifying evidence that loneliness is predictive of serious negative physical, cognitive, and psychological consequences.

One possible way to mitigate the effects of loneliness could be through internet-based social technology, which refers to online technology platforms that allow for real-time video, voice, and instant messaging communication between people. Internet-based social technology includes such platforms as Zoom, Skype, WhatsApp, or Facebook (6, 25). If social technology effectively mitigates feelings of loneliness, it may lead to improved health among older adults. Interventions aimed at leveraging social technology must consider the challenges in digital access among older adults. Specific barriers to technology use among older adults include the physical limitations in vision and motor function, anxiety and lack of confidence with technology, perceived lack of usefulness and usability, and technological designs that are not suited for older adults (26–33). Despite these challenges, overall, older adults tend to have favorable views of technology (34, 35). Over 75% of older adults report that they believe the Internet has been a positive commodity for them personally (35). Similarly, other work has observed that older adults perceive technology as a means to acquire information, strengthen family ties, increase social connectedness, and increase the quality and quantity of social communication (27, 36). Once older adults have access to technology, it appears that the perceived benefits offset perceived challenges to technology use.

Several studies have demonstrated that technology use can have positive psychosocial impacts. Internet and technology use among older adults is associated with greater life satisfaction, and subjective well-being, decreased depressive symptoms, greater social engagement, and more social support (10, 34, 37–42). Numerous studies found that using the Internet for social communication purposes is associated with lower levels of loneliness (6, 27, 36, 42–44). One study demonstrated that the relationship between social technology use frequency and decreased loneliness was mediated by perceived social support (6). Social technology use can be an effective tool to foster social support, which subsequently can decrease feelings of loneliness. Other findings show that loneliness mediates the relationship between heightened social technology use and physical and mental health; loneliness may therefore represent a psychological mechanism that explains how social technologies can enhance older adults' health (34). This body of research establishes strong evidence that social technology use among older adults can be beneficial in developing and maintaining meaningful, supportive relationships.

However, there may be disparities in the relationship between loneliness and social technology use. Black/African-American and other racial and ethnic minority older adults tend to have less equitable access to health and social service resources compared to White/Caucasian older adults [e.g., (45–48)]. Prior research focused on Black/African American populations has demonstrated that support from social networks may mitigate these barriers (47, 49, 50). Given this past research showing that supportive social networks may be particularly beneficial for Black/African-American racial minorities, the present study focuses specifically on racial differences in terms of Black/African-Americans and White/Caucasians. Research aimed at examining racial differences between Blacks/African Americans and Whites/Caucasians in loneliness and social isolation present mixed findings. Some work finds that Black older adults have smaller social networks, lower levels of social interaction, and greater levels of social isolation overall compared to White older adults (51, 52). An analysis using a demographic microsimulation model projected a doubling in the numbers of White kinless older adults by 2060, with a concomitant tripling among older Blacks over the same period (53). Socioeconomic disadvantage and health disparities among Black Americans contribute to an overall lifespan that is, on average, 3.5 years shorter than White Americans (54). Thus, loss of kin relationships into late adulthood is more likely to occur because family members, such as siblings, have higher rates of early mortality (53). Because kin are often a source of social support, racial inequalities in the burden of declining kin may disproportionately decrease social support and magnify the problem of loneliness among Black older adults in the future. However, other studies have not observed racial differences in loneliness (55) or social isolation (56–58). The majority of these studies have utilized large-scale, representative U.S. samples with similar outcome measures. Nevertheless, taken together, the inconclusiveness of this work underscores the need for further research to better understand racial differences in loneliness.

Similar to the relationship between race/ethnicity and loneliness findings, research that has sought to characterize rural-urban differences in loneliness also portrays complex, inconsistent results. On the one hand, several large-scale epidemiological studies in the United States (U.S.) and Canada have observed no association between rural-urban residence and loneliness (59–62). In contrast, other longitudinal research with U.S. populations shows that rural older adults report being able to rely on friends and family more than urban older adults (63). However, rural Black older adults report significantly higher levels of loneliness than other groups (63), suggesting that interactions between race and rurality may influence loneliness. Beyond this study, little research has examined how the interplay between race and rurality influences feelings of loneliness.

Although there is a strong link between social technology use and decreased loneliness in older adults, it is unclear whether there are racial or rural disparities in this relationship. Using data from the Health and Retirement Study, the present study examines whether the relationship between social technology use and loneliness differs by race and geographic region. Building on previous research, we propose the following hypotheses:

Hypothesis #1: Rural-dwelling older adults will report less social technology use compared to urban-dwelling older adults.
Hypothesis #2: Older Black adults will report less social technology use compared to older White adults.
Hypothesis #3: There will be a negative relationship between social technology use and loneliness such that lower social technology use will predict higher levels of loneliness.
Hypothesis 3a: This negative relationship is expected to be larger in magnitude among Black older adults compared to White older adults.
Hypothesis 3b: This negative relationship is expected to be larger in magnitude among rural-dwelling compared to urban-dwelling older adults.

## Method

### Data Source and Study Sample

The current study received ethics approval from Clemson University's Institutional Review Board before data acquisition. The data source for this research is the Health and Retirement Study (HRS), a nationally representative longitudinal study of Americans aged 50 and older that includes demographics, health, and cognitive measures. The HRS is an ongoing study conducted by the Institute for Social Research at the University of Michigan that was launched in 1992 (64, 65). Participants are surveyed in waves every 2 years. In 2006, the HRS introduced the Participant Psychosocial and Lifestyle Questionnaire (also called the “Leave-Behind” Questionnaire) that assesses numerous dimensions of psychosocial functioning (66). A subsample (50%) of respondents completes this survey during every biannual survey wave. The present study used data from the 2016 Core and Psychosocial and Lifestyle Questionnaire wave for all variables except the Beale-rural-urban continuum codes, which were not surveyed in 2016. Instead, Beale rural-urban continuum codes for the 2016 respondents were obtained by pooling data from the 2013 and 2003 HRS Cross-Wave Census Region/Division data waves (using the 2003 response if a 2013 response was missing).

### Measures

#### Demographics

Demographic information (age, marital living arrangements, gender, and race) was retrieved from the 2016 Core and “Leave-Behind” Questionnaire datasets. Age was obtained from the participant's reported date of birth, which was then subtracted from the year they completed the survey (2016). Marital living arrangements indicate whether the participant had a spouse or partner with whom they live. Gender was dichotomized as male and female, and race was defined as non-Hispanic White/Caucasian, non-Hispanic Black/African American, American Indian/Alaskan Native, Asian, Hawaiian Native/Pacific Islander, and other.

#### Beale Rural-Urban Continuum Codes

The U.S. Department of Agriculture (USDA) developed this nine-category classification system to categorize counties based on their degree of metropolitan vs. non-metropolitan characteristics (67). In the HRS Dataset, these nine categories are grouped into three clusters: continuum code of 1 is categorized as urban (metropolitan areas with population > 1,000,000), continuum code of 2 is categorized as suburban (metropolitan counties with a population of 250,000–1,000,000), and continuum code of 3–9 is categorized as rural (non-metropolitan counties with a population <250,000).

#### Social Technology Use

Following the approach from a recent study using the HRS dataset (6), social technology use was measured based on separate questions assessing self-reported frequency of social technology communication with children, other family members, and friends. Participants were asked “On average, how often do you communicate by Skype, Facebook, or other social media with any of your (children, other family members, friends) not counting any who live with you?” These three items were averaged such that higher scores reflect higher social technology use with family and friends. This measure was validated with older adults in previous research and showed high internal consistency [α = 0.87; (6)].

#### Loneliness

The 11-item version of the Revised UCLA Loneliness Scale (68) was used to measure subjective feelings of loneliness and social isolation. Sample items include the frequency with which participants feel “part of a group of friends,” “isolated (reverse-scored),” and “alone (reverse-coded)” using a 3-point Likert scale (α = 0.88). This scale version has been validated in older adult populations and showed high internal consistency [α = 0.87; (69)]. Sum scores were computed such that higher scores reflect higher levels of loneliness.

#### Perceived Social Support

The perceived social support items measure supportive relationships with family and friends (70, 71). Perceived social support was assessed through 12 separate questions regarding how well the participant feels their partner/spouse, children, other family members, and friends (a) understand the way they feel, (b) can be relied upon if they have a serious problem, and (c) they can open up to and talk about their worries based on a 4-point Likert scale (α = 0.81). Higher scores indicate greater average levels of perceived social support.

#### Perceived Social Negativity

The perceived social negativity items measure strained relationship interactions with family and friends (70, 71). Participants responded to 16 separate questions about their perception of how their partner/spouse, children, other family members, and friends (a) make too many demands on the participant, (b) criticize the participant, (c) let the participant down when the participant is counting on them, and (d) get on the participant's nerves using a 4-point Likert scale (α = 0.86). Higher scores reflect greater average levels of perceived social negativity.

#### Social Engagement

Social engagement, an index of social isolation, is defined as voluntarily participating in social activities. In line with previous research (72), social engagement was operationalized as the frequency of engagement in the following seven activities using a 7-point Likert scale (α = 0.66): (1) work with children or young people, (2) do activities with grandchildren, nieces/nephews, or neighborhood children, (3) volunteer, (4) attend educational or training courses, (5) go to a sport, social, or other club, (6), participate in a local community arts group such as choir, dance, etc., and (7) attend meetings of non-religious organizations, such as political or community groups. Higher scores indicate more social engagement on average.

#### Social Contact

Social contact, a social isolation metric, was assessed through nine total items that asked about the frequency with which participants (a) meet up, (b) talk on the phone, or (c) write/email with their children, other family members, or friends using a 6-point Likert scale [α = 0.71; (6)]. Higher values are indicative of greater social contact with family and friends.

#### General Computer Usage

A measure of participants' general computer usage was included as a covariate. Participants indicated the frequency in which they used a computer for email, Internet, or other tasks on a 7-point scale. Higher numbers indicate greater general computer usage.

### Data Analysis

The categorical variable of race was categorized as non-Hispanic Black/African-American, other racial/ethnic group (including Asian, American Indian/Alaskan Native, and Native Hawaiian/Pacific Islander, and individuals who identified as “other”), or non-Hispanic White/Caucasian. The categorical variable rurality was operationalized as rural, suburban, or urban. Correlations were first performed to examine the bivariate relationships among continuous study variables: social technology use, loneliness, perceived social support, perceived social negativity, social contact, social engagement, and age. To test the hypothesis that rural-dwelling (Hypothesis 1) and Black (Hypothesis 2) older adults will report less social technology use, a two-way factorial ANOVA comparing differences in social technology use by race and rurality was performed.

**Figure 1:**
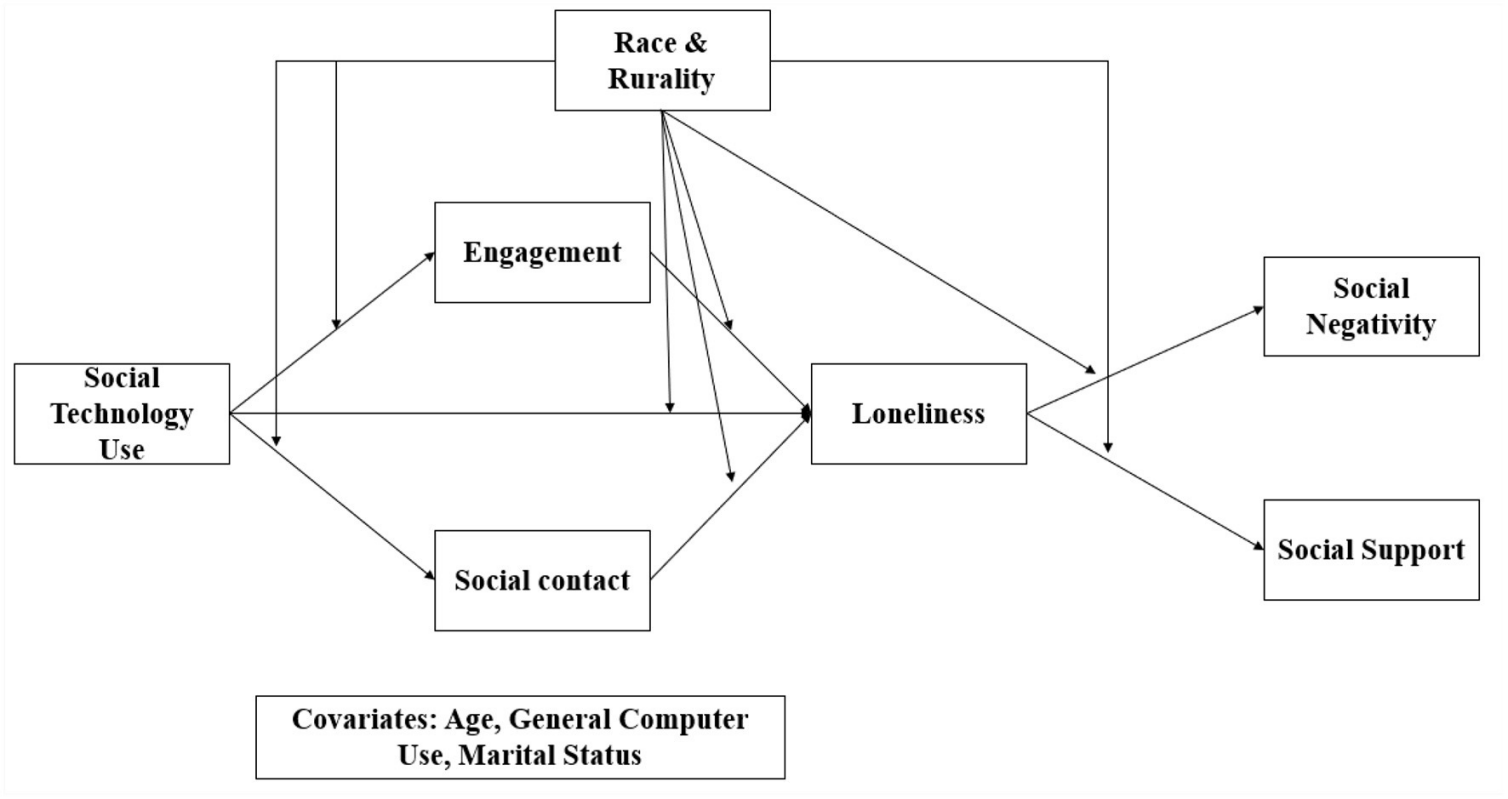
Conceptual model of the relationship between social technology use and loneliness. Race (White/Caucasian, Black/African American, and members of other racial/ethnic backgrounds) and rurality (rural suburban, and urban) were examined as moderators. Social engagement and social contacts were included as mediators of this putative relationship. Age, marital living arrangements (live with spouse/partner vs. not), and frequency of general computer usage were included as covariates in the model.

To examine the effects of race, rurality, and social technology use on loneliness (Hypothesis 3), a path model was performed (Figure 1). The social isolation metrics of social engagement and social contacts were included as mediators as these constructs related to social isolation. Sum scores of the constructs were used in the path model. To illustrate potential broader, downstream consequences of social technology use, perceived social negativity and perceived social support were also included in the model. The covariates marital living arrangements, age, and general computer usage were also included in the model. In order to enhance the robustness of the model, we used a maximum likelihood estimation with robust standard errors (“mlr”) and used the “lavaan” package in R to conduct the analyses.

## Results

### Sample Demographics

After pooling data across waves and excluding participants who reported being younger than age 50 at the time of responding (*n* = 68), the dataset used for the path model analysis contained 4,315 observations without missing data (*M*_*age*_ = 69.79, *SD*_*age*_ = 9.86, 60.6% female). For analytic purposes, we stratified race as non-Hispanic White/Caucasian (76.64% of the sample), non-Hispanic Black/African-American (16.01% of the sample), and members of other racial/ethnic backgrounds (7.26% of the sample). The other racial/ethnicity group was aggregated into a single category due to small sample sizes for each of these individual racial/ethnic groups [American Indian/Alaskan Native (*n* = 43), Asian (*n* = 57), Native Hawaiian/Pacific Islander (*n* = 7), and Other (*n* = 210)]. Within the other racial/ethnic group category, 210 participants identified as “other” which may include some individuals who self-identify as Hispanic/Latino as well as multiracial individuals. We note that for the ANOVA (*N* = 5,241) and ancillary correlations (*N* = 5,178), the sample contained more than the number of observations for the path model analysis. Demographic information for the path model analysis is shown in Table 1.

**Table 1:** Demographic variables (*N* = 4,315).

**Variable**	**Mean**	**Standard deviation**
**Age**	69.79	9.86
**Variable**	***n***	**%**
**Gender**		
Female	2,616	60.6%
Male	1,699	39.4%
**Race**		
Non-Hispanic White/Caucasian	3,306	76.64%
Non-Hispanic Black/African-American	692	16.01%
Other racial/ethnic backgrounds	317	7.26%
**Marital living arrangement**		
Lives with spouse	2,674	62.0%
Does not live with spouse	1,641	38.0%
**Beale codes**		
Urban	2,241	51.9%
Suburban	958	22.2%
Ex-urban/rural	1,116	25.9%

### Measures of Social Technology Use, Loneliness, and Social Isolation

The average social technology use across all participants was 2.46 (*SD* = 1.70, range = 1–6) on the 6-point scale. Using the response scale, this mean value reflects an average social technology use between “once or twice a year” and “every few months.” A Kruskal-Wallis one-way ANOVA demonstrated social technology use differences by rurality (*p* < 0.001) but not by race (*p* = 0.47) such that rural-dwelling older adults reported less social technology use than suburban or urban-dwelling older adults. Table 2 shows additional descriptive information for the continuous variables stratified by race and rurality.

**Table 2:** Descriptive information for primary study variables stratified by race and rurality (*N* = 4,315).

**Stratification by race**						
**Variable**	**Score range**	**Black/African American (** ***N*** **=** **692)**	**Other racial group (** ***N*** **=** **317)**	**White/Caucasian (** ***N*** **=** **3,306)**	**Total**	***P*** **-value** ^**a**^
	**Range**	**Mean (SD)**	**Mean (SD)**	**Mean (SD)**	**Mean (SD)**	
Social technology use	1–6	2.47 (1.73)	2.58 (1.74)	2.47 (1.69)	2.48 (1.70)	0.47
Loneliness	11–33	17.11 (4.80)	17.65 (4.81)	16.59 (4.79)	16.75 (4.80)	<0.001^*^
Social contact	1–6	3.61 (0.89)	3.62 (0.93)	3.72 (0.85)	3.69 (0.86)	0.002^*^
Social engagement	1–7	2.07 (0.86)	1.94 (0.84)	1.99 (0.77)	2.00 (0.79)	0.08
Social support	1–4	3.15 (0.56)	3.12 (0.56)	3.14 (0.54)	3.14 (0.54)	0.55
Social negativity	1–4	1.72 (0.54)	1.76 (0.56)	1.60 (0.44)	1.63 (0.47)	<0.001^*^
General computer use	1–7	4.43 (2.66)	4.52 (2.64)	5.10 (2.57)	4.95 (2.61)	<0.001^*^
**Stratification by rurality**						
**Variable**	**Score range**	**Rural (** ***N*** **=** **1,116)**	**Suburban (** ***N*** **=** **958)**	**Urban (** ***N*** **=** **2,241)**	**Total**	***P*** **-value** ^a^
	**Range**	**Mean (SD)**	**Mean (SD)**	**Mean (SD)**	**Mean (SD)**	
Social technology use	1–6	2.32 (1.66)	2.54 (1.72)	2.53 (1.71)	2.48 (1.70)	0.001^*^
Loneliness	11–33	17.06 (4.76)	16.87 (4.89)	16.55 (4.78)	16.75 (4.80)	0.003^*^
Social contact	1–6	3.58 (0.82)	3.72 (0.86)	3.74 (0.88)	3.69 (0.86)	<0.001^*^
Social engagement	1–7	1.97 (0.76)	2.00 (0.82)	2.02 (0.79)	2.00 (0.79)	0.23
Social support	1–4	3.12 (0.55)	3.15 (0.54)	3.15 (0.54)	3.14 (0.54)	0.20
Social negativity	1–4	1.61 (0.45)	1.63 (0.49)	1.64 (0.48)	1.63 (0.47)	0.40
General computer usage	1–7	4.43 (2.77)	4.86 (2.62)	5.24 (2.47)	4.95 (2.61)	<0.001^*^

a *Indicates the results of a Kruskal-Wallis test*.

* *indicates significance at the p < 0.05 level*.

### Relationships Between Social Technology Use, Loneliness, and Social Isolation Measures

Results revealed significant correlations among almost all continuous variables. Social technology use showed a significant moderate positive association with frequency of social contact (*r* = 0.47, *p* < 0.01), and significant but weak positive association with perceived social support (*r* = 0.16, *p* < 0.01) and frequency of social engagement (*r* = 0.22, *p* < 0.01). Furthermore, there was a significant moderate negative relationship between social technology use and age (*r* = −0.33, *p* < 0.01), and significant negative, albeit weak, association between social technology use and loneliness (*r* = −0.12, *p* < 0.01). Table 3 shows the correlations among the other continuous study variables.

**Table 3:** Correlational analyses (*N* = 5,178).

**Variable**	**Social tech. use**	**Loneliness**	**Social support**	**Social negativity**	**Social engagement**	**Social contact**
1. Social technology use						
2. Loneliness	−0.12^**^					
3. Social support	0.16^**^	−0.51^**^				
4. Social negativity	0.07	0.36^**^	−0.34^**^			
5. Social engagement	0.22^**^	−0.19^**^	0.10^**^	0.08^**^		
6. Social contact	0.47^**^	−0.30^**^	0.41^**^	−0.07^**^	0.33^**^	
7. Age	−0.33^**^	−0.02	0.05^**^	−0.22^**^	−0.14^**^	−0.08^**^

** *indicates significance at the p < 0.01 level*.

### Two-Way Factorial ANOVA Comparing Differences in Social Technology Use

The ANOVA examining differences in social technology use by race and rurality showed a significant main effect of rurality (*p* = 0.014) such that rural older adults (*M* = 2.32, *SD* = 1.66) reported significantly lower social technology use than older adults living in urban (*M* = 2.53, *SD* = 1.71) and suburban (*M* = 2.54, *SD* = 1.72) regions. There was no main effect of race (*p* = 0.55) or interaction (*p* = 0.40). Table 4 shows the ANOVA results.

**Table 4:** Two-way factorial analysis of variance results on the effect of race and rurality on differences in social technology use (*N* = 4,315).

**Source of variance**	**Degrees of freedom**	***F***	***p***
Race	2	0.60	0.55
Rurality	2	4.28	0.01^*^
Race^*^rurality interaction	4	1.01	0.40
Error	4,306	2.89	

*Levels of race include Black/African-American, members of other racial/ethnic backgrounds, and White/Caucasian. Levels of Rurality include urban, suburban, and rural*.

* *indicates significance at the p < 0.05 level*.

### Path Model Analysis

#### Mediation Effects

**Figure 2:**
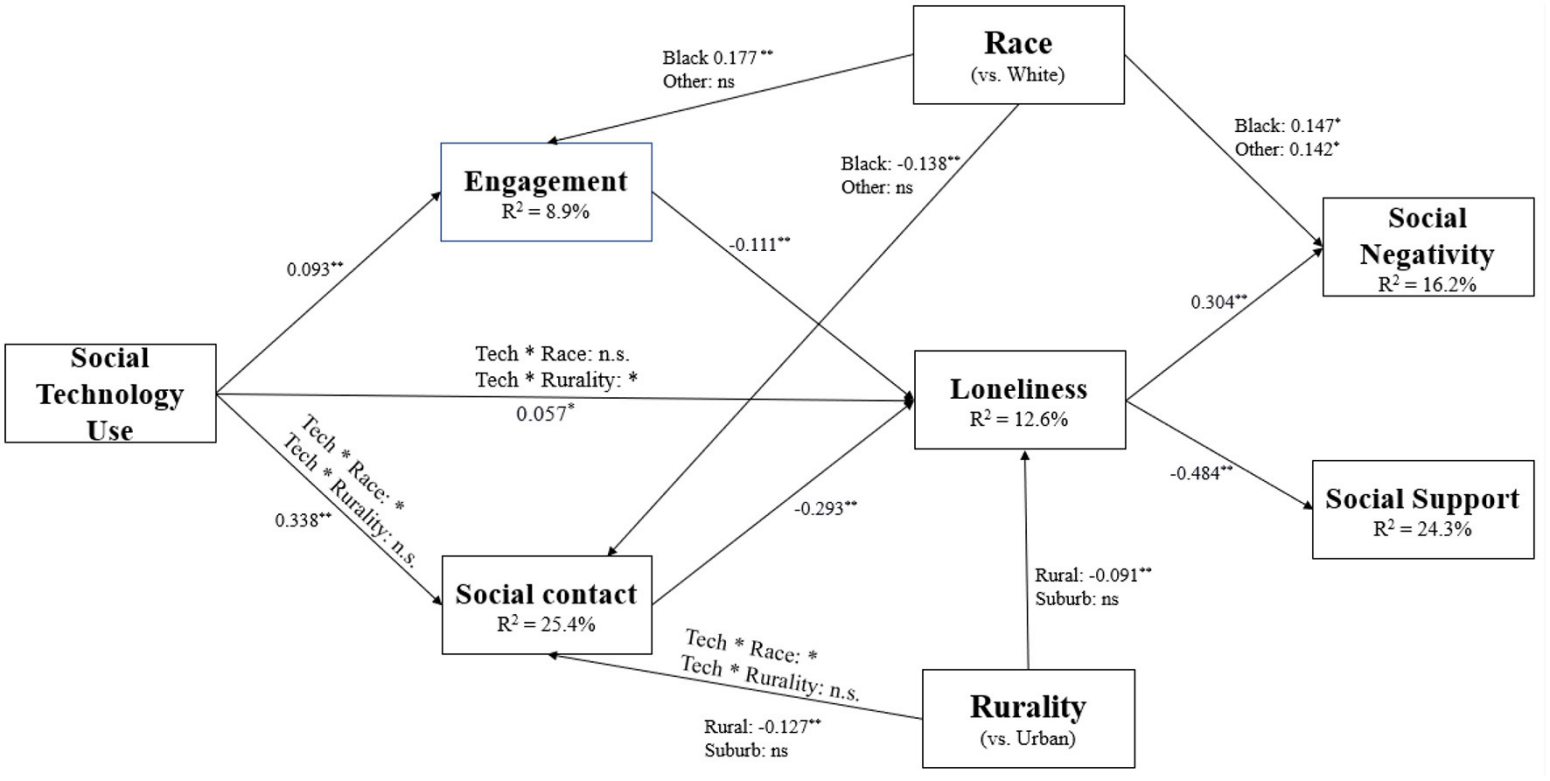
Path model results (*N* = 4,315) of the path model with social technology use predicting loneliness as moderated by race and rurality and mediated by social engagement and social contacts. The figure shows direct paths between variables. Path parameters represent standardized coefficients. *indicates *p* < 0.01. **indicates *p* < 0.001.

The results of the path model showed that the effect of social technology use on loneliness was partially mediated by social contact and social engagement. Table 5 shows the direct and indirect effects of the mediation analyses. Despite the direct positive main effect of social technology use on loneliness, using social technology increases social contact and social engagement and overall mitigates loneliness. Results of the full model are shown in Figure 2.

**Table 5:** Effect of social technology on loneliness as mediated by social contact and social engagement.

**Paths**	**Estimate**	**S.E**.	***p*-value**
**Direct path**			
Social technology use → loneliness	0.057	0.027	0.034
**Indirect paths**			
Social technology use → social contact → loneliness	−0.114	0.013	<0.001
Social technology use → social engagement → loneliness	−0.010	0.004	0.005
Total effect	−0.067	0.026	0.010

*S.E. refers to standard error*.

#### Loneliness

**Figure 3:**
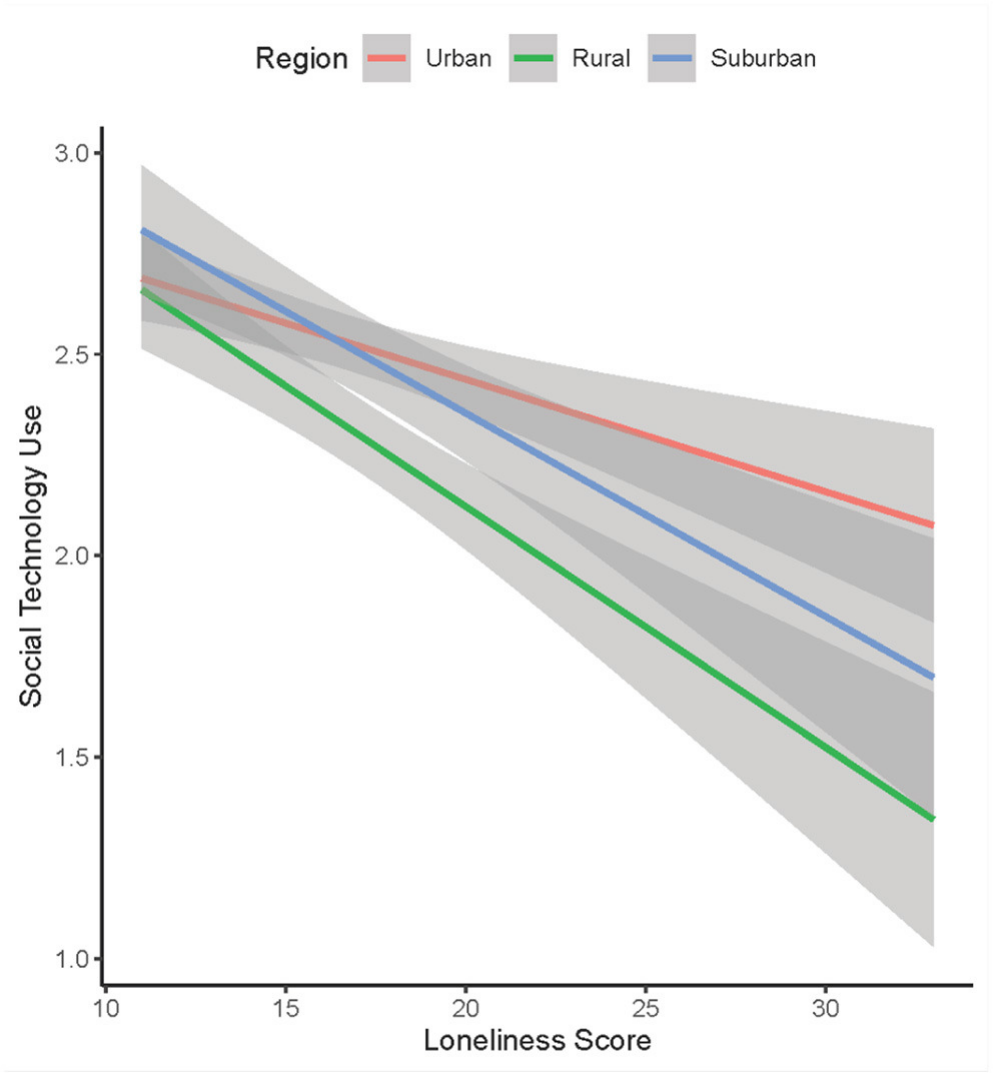
Relationship between loneliness and social technology use by rurality. The magnitude of the negative relationship between loneliness and social technology use was stronger among rural older adults than urban older adults.

Both social technology use ^*^ race and social technology use ^*^ rurality two-way interactions and three-way interaction effects between race, rurality, and social technology use were tested. The three-way interaction effects were not significant. The primary study result is reflected by a significant two-way interaction between rurality and social technology use (*p* omnibus = 0.034). This effect was localized to rural older adults (rural: β = −0.106, *p* = 0.011; suburb: β = −0.059, *p* = 0.187) such that rural older adults who use social technology more often reported higher levels of loneliness compared to urban-dwelling older adults (Figure 3). Results also revealed a main effect of rurality on loneliness (*p* omnibus = 0.041). Specifically, rural residents reported significantly higher levels of loneliness compared to urban residents (rural: β = 0.091, *p* = 0.018; suburb: β = 0.070, *p* = 0.093). The main effect of race on social technology use was not significant (*p* omnibus = 0.093). Furthermore, individuals with greater social engagement reported lower levels of loneliness (β = −0.111, *p* < 0.001). The direct effect of social technology use also significantly predicted loneliness (β = 0.057, *p* = 0.034). No other effects were significant. The model accounts for 12.6% of the variance in loneliness.

#### Social Engagement

Path models results with social technology use, race, rurality, and the covariates predicting social engagement revealed a significant main effect of race on social engagement (*p* omnibus = 0.003). Black older adults reported higher levels of social engagement than White older adults (β = 0.177, *p* = 0.001). Greater social technology use (β = 0.093, *p* < 0.001), lower levels of general computer usage (β = −0.073, *p* < 0.001), and living with a partner predicted higher social engagement. No other significant effects were observed.

#### Social Contact

Omnibus test results for frequency of social contact with family and friends suggested a significant interaction between social technology use and race (*p* omnibus = 0.048); members of other racial/ethnic backgrounds, including Asian, American Indian/Alaskan Native, Native Hawaiian/Pacific Islander, and those self-identifying as “other,” who use social technology more have greater social contact (β = 0.119, *p* = 0.021). A significant main effect of race on social contact (*p* omnibus = 0.011) showed that Black older adults reported significantly less contact with family and friends than White older adults (β = −0.138, *p* = 0.005). Rurality was also a significant predictor of social contact (*p* omnibus < 0.001) such that those living in rural regions reported significantly less social contact compared to those living in urban areas (β = −0.127, *p* < 0.001). Greater social technology use is also associated with greater social contact (β = 0.338, *p* < 0.001). Less frequent general computer use (β = −0.077, *p* < 0.001), older age (β = 0.010, *p* < 0.001) and living without a partner (β = −0.131, *p* < 0.001) predicted greater social contact frequency.

#### Perceived Social Support

Model results showed that loneliness was predictive of diminished perceived social support (β = −0.484, *p* < 0.001). Living with a partner was also associated with lower social support (β = −0.085, *p* < 0.01). No other significant effects were observed.

#### Perceived Social Negativity

A significant main effect of race on perceived social negativity (*p* omnibus = 0.001) revealed that both Black older adults and members of other minority races reported experiencing greater perceived social negativity (βs = 0.147, 0.142, *p*s < 0.01). There was also a significant main effect of loneliness in which lonelier older adults perceived greater social negativity (β = 0.304, *p* < 0.001). Greater general computer usage (β = 0.021, *p* < 0.001), younger age (β = −0.020, *p* < 0.001), and living with a partner (β = 0.157, *p* < 0.001) was associated with greater perceived social negativity.

## Discussion

Previous research has shown that older adults who engage in more frequent online social communication tend to be less lonely (6, 27, 36, 42–44). Correlational results from the present study are largely consistent with this work; greater frequency of internet-based social technology use was associated with lower levels of loneliness among older adults. While this result supports our hypothesis, we note that the observed strength of this relationship was relatively weak. Furthermore, internet-based social technology use and was associated with greater perceived social support and lower levels of social isolation, as measured by frequency of social contact and social engagement. These findings suggest that internet-based social technology use may present a tool to foster social support and connectedness among older adults.

The results of this study extends previous research on loneliness and social technology use by showing that the association between social technology use and loneliness is mediated by frequency of social engagement and social contact with friends and family; these mediators align with social isolation constructs. Although the direct effect of social technology on loneliness was positive, in the context of these mediators, the total effect of social technology use, mediated by social engagement and social contact, predicted lower levels of loneliness. Therefore, in addition to the observed rural disparity finding, this study provides a putative mechanism for the relationship between social technology use and loneliness: social technology use predicts increased frequency of social engagement and contact with family and friends, which in turn is predictive of reduced feelings of loneliness.

The present study also investigated racial and rural differences in the relationship between social technology use and loneliness. We predicted that there would be a negative relationship between loneliness and social technology use that would be exacerbated among Black and rural-dwelling older adults. The primary findings demonstrate that the association between loneliness and social technology use differed by rurality but *not* by race. Rural older adults who use social technology less frequently experienced higher levels of loneliness than urban older adults.

In addition to these findings, we further hypothesized that social technology use would be less prevalent among Black and rural older adults. The data supported the hypothesis for rurality but not the hypothesis for racial differences in social technology use. This finding is consistent with studies showing that individuals living in rural areas are less likely to use online technology than those in urban regions (73). Although rural older adults use social technology less than urban older adults, the benefits of technology in fostering social connectedness have previously been observed in rural communities (74). Moreover, the mediation results of the present study bolster these findings by showing that social technology use is associated with increased social engagement and contact with family and friends. Rural regions often have fewer central gathering places and opportunities to interact with neighbors, which can increase social isolation (10, 75). Social technology offers an alternative means for communication and socialization that can be capitalized on to reduce feelings of loneliness among older adults aged 50 and older. Given the rural disparities in technology use and the corresponding increase in loneliness, further research is needed to better understand the unique challenges rural older adults face in social technology utilization. Elucidating the major barriers to social technology use and implementing interventions to overcome these barriers among the at-risk rural older adults, such as technology training, adaptive interface designs for age-related decline, or hands-on services to deliver technology resources to rural regions, may be key to reducing loneliness and the associated health consequences of loneliness in this population.

Further analyses examining potential racial and rural differences in loneliness, social contact, social negativity, and social support were also performed. Rural individuals had fewer social contacts than urban individuals and experienced greater loneliness, which varies from prior studies that did not observe differences in rurality on loneliness (59–62). Differences in design methodology and sample demographics, including age and country of residence, may potentially contribute to these differences. Given the important consequences associated with loneliness, these findings underscore the need for further large-scale, longitudinal research that directly evaluates the impact and potential mechanisms of rural-urban differences in loneliness.

Results for racial differences showed that Black older adults had fewer social contacts and encountered more social negativity in their relationships than White older adults, although they had greater social engagement. These results are in line with prior research showing that Black older adults have smaller social networks compared to White older adults (51, 52). Study results did not show significant racial differences in loneliness, which supports prior research (55). The heightened social negativity among Black older adults is particularly concerning. The widespread discrimination that afflicts Black Americans is associated with increased risk of mortality and poor physical and psychological outcomes [e.g., (76–80)]. Merging the discrimination literature and the present study's findings, it appears that there may be compounding threats of social discrimination and social negativity from family and friends that disproportionately impact Black older adults. These threats pose serious health risks, and future research is needed to address the social inequalities that Black older adults encounter.

Moreover, study findings indicated that greater levels of loneliness were associated with significantly greater perceived social negativity and less perceived social support. This result echoes the conceptualization of loneliness as the subjective experience resulting from a dearth of supportive, meaningful relationships (7, 8). Loneliness among older adults appears to encompass the psychological experience of being burdened by draining social relationships that do not provide reliable support. Lack of social support can dampen psychological resources needed to adapt to age-related life changes and challenges, which can potentially compound health problems (24).

### Limitations and Future Directions

In considering the limitations of this study, it should be noted that the study utilized a cross-sectional correlational design, and causal relationships between social technology use and loneliness cannot be established. As previous research has noted, a self-selection bias could influence this relationship such that individuals who are more open to using social technology experience lower levels of loneliness (6). Future work utilizing randomized controlled trials with social technology interventions or longitudinal designs are needed to establish a causal relationship between social technology use and loneliness and the corresponding rural disparities in this relationship.

Moreover, in this study, social technology use was defined as the frequency of communication using Skype, Facebook, or other social media with friends and family. It is unclear which social technology platforms are particularly beneficial, and a fine-grain examination of the platforms and platform features that foster social benefits among older adults may be useful in identifying ways to increase social technology use in this population. Similarly, the reasons that rural older adults who use social technology less often tend to be more lonely than urban older adults were not explored as part of this investigation. Future research identifying the mechanisms of rural disparities in the relationship between loneliness and social technology use needs to be established.

We also note that the present investigation merged non-White/Caucasian and non-Black/African-American participants from other racial and ethnic groups into a single category due to sample size constraints. The results of this study cannot be generalized beyond White/Caucasian and Black/African-American groups, and it is unclear how the relationship between loneliness and social technology use may differ among Hispanic/Latino ethnic groups or Asian, Native American, or Pacific Islander racial groups. Additional research is needed to better characterize ways to reduce loneliness is these ethnic and racial groups.

### Conclusions

This work sought to characterize rural and racial disparities in the association between loneliness and online social communication frequency. This study provides evidence of rural, but not racial, disparities in the link between social technology and loneliness. Low social technology use is associated with greater loneliness among rural older adults compared to urban older adults. One potential implication from this work is that the benefits of social technology may be particularly impactful for rural older adults in combatting loneliness. Methods to increase social technology use among rural older adults may be beneficial in reducing loneliness and could, in turn, help alleviate the detrimental health consequences associated with loneliness.

## Data Availability Statement

Publicly available datasets were analyzed in this study. This data can be found here: https://hrs.isr.umich.edu/.

## Ethics Statement

This study involving secondary data analysis of research with human participants was reviewed and approved by Clemson University Institutional Review Board. Participants were part of the Health and Retirement Study (HRS) and provided their written informed consent to participate in the HRS study.

## Author Contributions

KB and SL contributed to the conceptualization of the study. RA performed data extraction, organization, and statistical analyses. BK provided guidance on the statistics. KB wrote the first draft of the manuscript. KC, CD, LR, BK, and SL reviewed the manuscript and provide critical feedback. All authors contributed to the manuscript revision, read, and approved the submitted version.

## Conflict of Interest

The authors declare that the research was conducted in the absence of any commercial or financial relationships that could be construed as a potential conflict of interest.

## Publisher's Note

All claims expressed in this article are solely those of the authors and do not necessarily represent those of their affiliated organizations, or those of the publisher, the editors and the reviewers. Any product that may be evaluated in this article, or claim that may be made by its manufacturer, is not guaranteed or endorsed by the publisher.
